# *Bacillus subtilis* Supplementation in Weanling Piglets: A Systematic Review of Growth, Gut Health, and Microbiota Modulation

**DOI:** 10.3390/ani16132054

**Published:** 2026-07-03

**Authors:** Charlotte Ludorf, Carley Richardson, Kwangwook Kim

**Affiliations:** Department of Animal Science, Michigan State University, East Lansing, MI 48824, USA; ludorfch@msu.edu (C.L.); rich1421@msu.edu (C.R.)

**Keywords:** *Bacillus subtilis*, direct-fed microbial, gut health, microbiota, piglet, probiotic, swine nutrition, weaning

## Abstract

*Bacillus subtilis* has been widely investigated as a probiotic feed additive for weanling piglets due to its potential to improve growth performance, gut health, and disease resilience during the post-weaning period. This systematic review evaluated the effects of *Bacillus subtilis* supplementation in pigs following PRISMA guidelines and the PICOS framework. A total of 619 records published between 2000 and 2025 were identified through PubMed, Scopus, and AGRICOLA databases, of which 29 studies met the inclusion criteria for qualitative synthesis. Overall, *Bacillus subtilis* supplementation frequently improved average daily gain, body weight, feed efficiency, and reduced diarrhea incidence, particularly under enteric challenge conditions. Many studies also reported beneficial effects on intestinal morphology, immune regulation, intestinal barrier-related gene expression, nutrient digestibility, and modulation of gut microbiota toward bacterial communities associated with intestinal health. However, responses varied depending on *Bacillus subtilis* strain, dosage, diet composition, and experimental design. In contrast, effects on biochemical and oxidative stress indicators were less consistent across studies. Collectively, the findings support the potential role of *Bacillus subtilis* as a functional probiotic in swine nutrition while highlighting the need for standardized long-term studies to better define optimal strains, supplementation strategies, and mechanisms of action.

## Simple Summary

This systematic review summarized published studies evaluating *Bacillus subtilis*, a beneficial bacterium used as a probiotic feed additive, in weanling piglets. Overall, many studies showed that *Bacillus subtilis* supplementation improved growth, reduced diarrhea, supported gut development, and increased the abundance of beneficial bacteria in the digestive tract. Some studies also reported improved immune responses and better protection of the intestinal lining, especially when piglets were exposed to disease or toxin challenges. However, results were not always consistent, likely because studies used different bacterial strains, supplementation levels, diets, and experimental conditions. The effects on indicators of metabolism and the piglets’ ability to cope with physiological stress were particularly variable among studies. These findings suggest that *Bacillus subtilis* may be a useful nutritional tool to support piglet health and productivity after weaning, but more long-term and standardized research is needed to identify the most effective strains, dosages, and feeding strategies.

## 1. Introduction

*Bacillus subtilis* is a Gram-positive, spore-forming bacterium that is widely used as a direct-fed microbial and probiotic in animal nutrition. Probiotics are live microorganisms that, when administered in adequate amounts, confer health benefits to the host, while direct-fed microbials are live microbial feed supplements intended to improve animal performance and health [1]. *Bacillus subtilis* has long been used as a model organism in biological research due to its natural transformability, rapid growth rate, and ease of cultivation [2]. As one of the most extensively studied Gram-positive bacteria, *Bacillus subtilis* has contributed substantially to advances in microbial genetics, physiology, and host–microbe interaction [3,4].

In recent decades, *Bacillus subtilis* has gained increasing attention as a dietary supplement in human and livestock nutrition, largely because of its high stability, resistance to harsh environmental conditions, and ability to survive feed processing and long-term storage [4,5,6]. As its application has expanded within livestock systems, particular interest has focused on its use during early life stages, when animals are most vulnerable to environmental stressors and gastrointestinal disturbances. Studies in ruminant species, including dairy calves, lambs, and goats, have reported positive effects of *Bacillus subtilis* supplementation on growth performance, health status, and gut microbial communities [7,8,9]. Similarly, research in non-ruminant species has demonstrated improvements in growth performance, intestinal development, and immune responses in broiler chickens receiving *Bacillus subtilis* supplementation [10,11,12,13,14].

Despite these generally favorable outcomes, the biological mechanisms underlying the beneficial effects of *Bacillus subtilis* supplementation in young livestock remain incompletely understood. Proposed mechanisms include modulation of gut microbial communities through the promotion of beneficial bacteria and suppression of harmful microorganisms, enhancement of intestinal development through improved villus height-to-crypt depth ratio to support nutrient absorption, and regulation of immune and antioxidant responses that may reduce inflammation [15,16]. A clearer understanding of these mechanisms is especially important in weanling piglets, as weaning is a critical production stage characterized by abrupt dietary, environmental, microbial, and immunological changes.

Previous reviews have discussed probiotics or *Bacillus subtilis* in swine nutrition; however, many have focused broadly on multiple probiotic species, general production stages, or livestock species. In addition, most existing reviews are narrative in nature and do not systematically compare how *Bacillus subtilis* strain, dose, diet composition, study duration, and disease challenge model influence outcomes. Therefore, although many individual studies have evaluated *Bacillus subtilis* supplementation in weanling piglets, uncertainty remains regarding which responses are most consistent, which biological mechanisms are best supported, and which factors explain the variability among studies.

Although a substantial number of studies have investigated *Bacillus subtilis* supplementation in weanling piglets, reported outcomes are highly variable. Many studies have documented improvements in growth performance, diarrhea incidence, intestinal morphology, immune parameters, and gut microbiota composition, whereas others report minimal or inconsistent effects. Differences in *Bacillus subtilis* strain, dosage, dietary composition, experimental design, study duration, and health challenge models likely contribute to this heterogeneity. As a result, a systematic synthesis is needed to clarify the magnitude and consistency of responses and to identify the conditions under which *Bacillus subtilis* supplementation is most likely to benefit piglet physiology, growth, and health.

Therefore, the objective of this systematic review is to provide a comprehensive synthesis of the existing literature evaluating the effects of *Bacillus subtilis* supplementation on growth performance, health-related outcomes, intestinal development, immune responses, and gut microbiome characteristics in weanling piglets. An additional aim is to critically assess the influence of strain selection, dosage, dietary context, study design, and challenge model on reported outcomes. By systematically integrating and comparing findings across studies, this review seeks to identify consistent effects, explain sources of heterogeneity, clarify potential mechanisms of action, and inform future research directions to optimize the practical application of *Bacillus subtilis* in pig nutrition.

## 2. Materials and Methods

### 2.1. Study Protocol

The protocol for this systematic review was registered on the Open Science Framework (OSF) Registries (ID: vx7d3) [17]. The systematic review was administered in accordance with the Preferred Reporting Items for Systematic Reviews and Meta-analysis (PRISMA) guidelines.

### 2.2. Eligibility Criteria

The summary of the inclusion and exclusion criteria of the study properties, established from the populations, interventions, comparators, outcomes of interest, and study designs (PICOS) framework, is presented in Table 1 [18]. Peer-reviewed papers that were available in full-text and written in English were included. Review papers, abstracts, protocols, editorials, opinion pieces, and dissertations were excluded.

### 2.3. Information Sources and Search Strategy

The published research studies in this systematic review were identified through searches in scientific databases using the PubMed Advanced Search Builder, Scopus, and AGRICOLA (USDA). Boolean operators were applied to a series of keywords to capture studies on *Bacillus subtilis* in pigs. Comprehensive search strategies for each database are presented in Table 2. All searches were conducted in February 2025, with a publication date range restricted to 2000 through 2025.

### 2.4. Study Selection

All retrieved references were imported into Covidence systematic review software [19], where duplicates records were removed. Prior to screening, two researchers (C.L. and C.R.) developed a standardized procedure for title and abstract screening, piloted using 10 randomly selected papers. In the first screening phase, C.L. and C.R. independently evaluated all titles and abstracts against the eligibility criteria outlined in Table 1. Subsequently, full-text articles were reviewed. Discrepancies at any stage were resolved through discussion; if consensus could not be reached, a third reviewer (K.K.) served as the arbitrator.

### 2.5. Data Collection Process and Data Items

Using predefined study criteria, data extraction was performed independently by two reviewers, C.L. and C.R., using a standardized data extraction table initially developed by C.L. and reviewed by K.K. For each study, the following information was recorded: authors; year of publication; country of study; number and species of animals; age or body weight at the start of the experiment; type and dosage of *Bacillus subtilis* administered; study duration; measured parameters, such as growth performance, feed intake, gut health, and biochemical markers; and key findings, including the effects of *Bacillus subtilis* on the evaluated outcomes. Discrepancies between reviewers were resolved through discussion, and K.K. verified the final extracted data to ensure accuracy and completeness. Data were systematically extracted to facilitate comparison and qualitative synthesis across studies.

### 2.6. Quality Assessment and Risk of Bias

Two reviewers (C.L. and C.R.) independently assessed the risk of bias in the studies, including using Systematic Review Centre for Laboratory Animal Experimentation (SYRCLE)’s risk of bias tool for animal experimental studies. The checklist comprises ten domains grouped into six categories of bias: sequence generation, baseline characteristics, and allocation concealment (selection bias); random housing and blinding of caregivers and/or investigators (performance bias); random outcome assessment and blinding of outcome assessors (detection bias); incomplete outcome data (attrition bias); selective outcome reporting (reporting bias); and other potential sources of bias. Each item was rated as “low risk of bias”, “high risk of bias”, or “unclear risk of bias”. Disagreements were resolved through discussion, and if consensus could not be reached, a third reviewer (K.K.) acted as the arbitrator.

### 2.7. Synthesis of Results

The feasibility of conducting a meta-analysis was assessed by comparing study designs, intervention characteristics, outcome measures, reporting formats, and availability of variance data across the included studies. Quantitative synthesis was not performed because of substantial heterogeneity among studies, including differences in *Bacillus subtilis* strain, dosage, supplementation duration, diet composition, piglet age, health challenge model, comparator groups, sampling time points, and outcome reporting. In addition, several studies did not report outcomes using comparable units or provide sufficient variance measures required for pooled effect estimates. Therefore, subgroup analyses or other quantitative approaches were not conducted, and a qualitative synthesis was performed to summarize and compare findings across studies.

## 3. Results and Discussion

### 3.1. Study Selection Process

**Figure 1:**
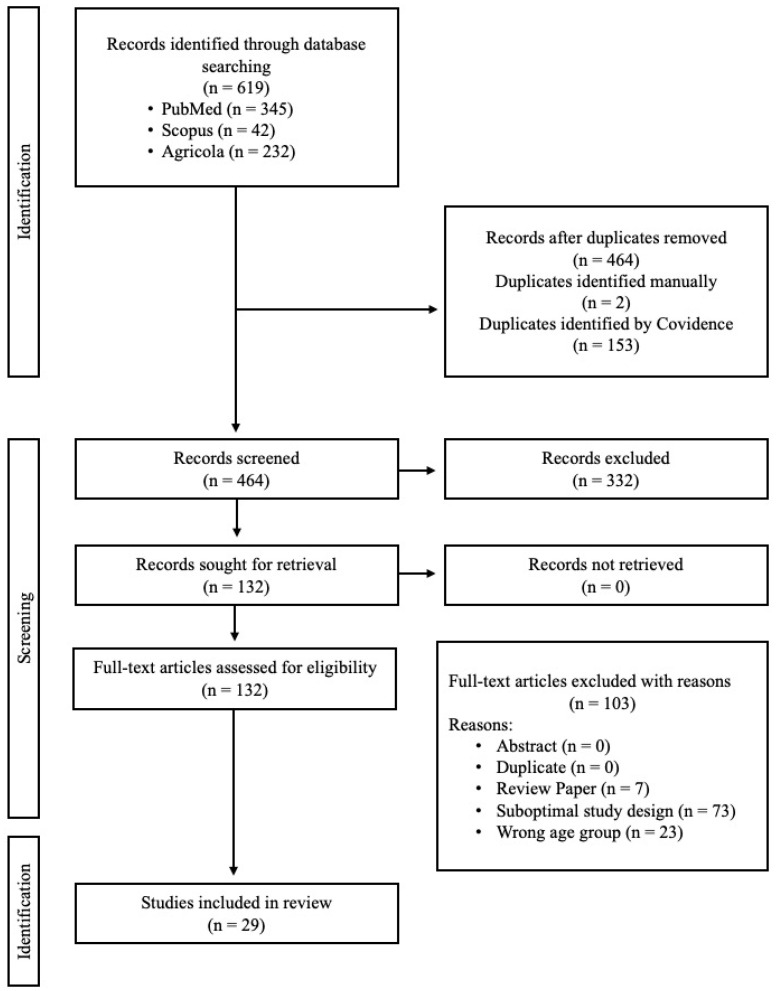
The PRISMA flow diagram using Covidence literature screening on *Bacillus subtilis* supplementation in post-weaning pigs.

Due to a large number of results being yielded from the original search, the papers were narrowed down to include the impact of *Bacillus subtilis* on post-weaning pigs. The study selection process is shown in Figure 1. The search yielded 619 references in total and from these references 153 duplicates were removed. In total, 464 abstracts were screened, with 332 being judged to be ineligible, ending with 132 papers to be read in full-text. A total of 29 papers met the eligibility criteria and were included in the systematic review.

### 3.2. Summary of Study Designs and Sample Characteristics

The characteristics of each study are provided in Table 3. There were 22 papers that were published between 2020 and 2024, and 7 papers that were published between 2010 and 2019. In the studies, the total sample size ranged from 12 pigs [20,21,22] to 360 pigs [23].

A majority of the studies utilized crossbred pigs as the sample population. The crossbreds included Duroc × Large White × Landrace [24,25], Large White × Landrace [26,27,28], Yorkshire × Landrace [21,22,29], Yorkshire × Landrace × Duroc [16,23,30,31,32,33,34,35], PIC 359 × Yorkshire [36], and PIC1050 × DNA600 [37]. One study used a mixture of breeds including Yorkshire × Yorkshire, Yorkshire × Duroc, and Yorkshire × Duroc × Duroc [38]. Ten of the studies did not include the breed [5,15,20,39,40,41,42,43,44,45].

Most of the studies used young pigs that ranged from 21 to 28 days of age or had a weight between 5.6 ± 0.3 kg to 9.1 ± 1.3 kg. One study used young pigs 45 days of age and had a body weight of 14.06 ± 1.80 kg [24]. One study did not include the days of age or initial body weights, and just specified that newborns were used [20].

**Table 3:** The summary of studies and main results of effects of *Bacillus subtilis* supplementation on post-weaning pigs.

Authors, Year, Country	Number of Animals/Species	Age	Type of *Bacillus subtilis*	Experiment Duration	Characteristics Evaluated	Key Effects of *Bacillus subtilis* Supplementation
Deng et al. [24], China	72, Duroc × Large White × Landrace	45 days; initial BW: 14.06 ± 1.80 kg	0.1% *Bacillus subtilis* (BF7658, CGMCC 1.240) 1 × 10^10^ cfu/g	28 days	Growth performance; Serum parameters; Mucosal digestive enzymes; Intestinal morphology; Colonic microbiota	↑ ADG; ↑ Serum triglycerides and lipase, amylase, maltase activities in the ileum; ↑ VH: CD in the ileum; ↑ Serum glucose in the ileum; ↑ Abundance of *Firmicutes* in the colonic microbiota; ↓ Abundance of *E. coli* in the colonic microbiota
Ding et al. [26], China	128 barrows, Large White × Landrace	21 days; initial BW: 7.84 ± 0.02 kg	500 g/t *Bacillus subtilis* (DSM 32315) with spore count of 2 × 10^9^ cfu/g	42 days	Intestinal morphology; Metabolite levels; Intestine microbiota diversity; Intestine microbiota composition	↑ VH and VH:CD in ileum; ↑ Colonic concentrations of butyrate, tryptamine, and cadaverine; ↓ Colonic skatole concentration; ↑ Gut microbial diversity and alterations in bacterial species abundance
Ding et al. [27], China	64, Large White × Landrace barrows	21 days; initial BW: N/A	500 g/t *Bacillus subtilis* (DSM 32315) with spore count of 2 × 10^9^ cfu/g	42 days	Intestinal morphology; Intestine metabolites; Intestine microbiota diversity and composition	↑ Abundance of jejunal *Leucobacter* and *Cupriavidus*; ↑ Abundance of ileal *Thermus*, *Coprococcus* and *Bifidobacterium*; ↑ Abundance of colonic *Succiniclasticum*; ↑ Ileal SCFA; ↑ Colonic propionate, branched-chain fatty acids, and tyramine concentrations; ↓ Colonic indole concentration
Duddeck et al. [38], United States	54, Yorkshire × Yorkshire or Yorkshire × Duroc, Yorkshire × Duroc × Duroc	26.9 ± 2.0 days; initial BW: 9.1 ± 1.3 kg	3.75 × 10^8^ cfu/g *Bacillus subtilis* (1.875 × 10^5^ cfu/g diet); 3.75 × 10^9^ cfu/g *Bacillus subtilis* (1.875 × 10^6^ cfu/g diet)	28 days	Growth performance; Fecal score; Blood parameters; Fecal SCFA concentrations; Fecal bile concentration; Fecal microbiome	↑ ADG and BW on day 14 with low dose; ↑ Blood glucose on day 14 with both dose; ↑ Fecal butyrate and isovalerate with low dose; ↑ Fecal acetate, propionate, and total SCFAs with low dose; ↑ Microbial evenness with low dose at day 28; ↑ Beta diversity difference between low and high dose at day 14;
Du et al. [20], China	12	Newborn; initial BW: N/A	5 × 10^9^ cfu of the *Bacillus* (WS-1; 1 mL of medium contained 1 × 10^9^ cfu)	6 days	Health status; Diarrhea rate; Histopathology; Genome sequencing	↓ Diarrhea rate on day 1 PI; ↓ Mortality rate
Garvey et al. [45], United States	32, crossbred	21 days; initial BW: 7.12 ± 1.70 kg	7.5 billion cfu/day *Bacillus subtilis* (MB40); 7.5 billion cfu/day *Bacillus subtilis* (MB40 with *Listeria monocytogenes* challenge)	14 days	Growth performance; Organ weights; Bacterial counts; Hematology; Plasma cytokines; Plasma biochemistry; Intestinal morphology;	↓ Listeria monocytogenes count in liver and spleen; ↓ Circulating monocyte concentrations
He et al. [22], China	12, Yorkshire × Landrace	28 days; initial BW: N/A	10 mL of *Bacillus subtilis* (1 × 10^9^ cfu/mL JATP3)	28 days	Intestinal morphology; Intestinal pH; Intestinal gene expression; Mucosa microorganisms; Intestinal mucosa microbial composition; Metabolic profile of chime	↑ VH:CD in jejunum and ileum ↑ *Claudin*, *ZO-1*, and *IL-10* expression in jejunum and ileum; ↓ *IL-1β* expression in jejunum and ileum; ↑ Beneficial microbial abundance in jejunum and ileum; ↑ Citalopram, isobutyric acid, and isocitric acid in ileum; ↑ Positive correlation between citalopram and microbial changes in jejunum and ileum
He et al. [21], China	12, Yorkshire × Landrace	28 days; initial BW: N/A	10 mL of *Bacillus subtilis* (1 × 10^9^ cfu/mL JATP3)	28 days	Growth performance; Nitrogen metabolism in urine, feces, and feed; Expression of proteins related to protein synthesis in the skeletal muscles; AKG content in liver and muscle; Metabolomic profile of portal vein plasma; Intestinal microbiome	↑ Final nitrogen deposition rate in the urine, feces, and feed; ↑ Abundance of *Pediococcus*, *Collinsella*, *Veillonella*, *Clostridium*, and *Escherichia* in jejunum; ↑ Abundance of *Olsenella* and *Pediococcus* in ileum; ↑ Portal vein L-lactic acid and AKG levels; ↑ AKG concentrations in muscle and liver; ↑ Phosphorylated 4EBP1 in skeletal muscle; ↑ Abundance of *Olsenella* and *Pediococcu* in ileum; ↓ Abundance of *Weissella* in ileum; ↑ L-lactic acid and AKG levels in the portal vein plasma; ↑ In the content of AKG levels in the muscle and liver
Herrera Franco et al. [42], Colombia	150 castrated male, commercial terminal crossing	21 days; initial BW: 5.6 ± 0.3 kg 28 days old; initial BW: ~6.8 ± 0.4 kg	50 ppm of *Bacillus subtilis* (PTA-6737; 10^8^ cfu)	30 days	Growth performance; Intestinal morphology; mRNA abundances in the intestinal barrier protein genes; Gene abundances in the jejunum tight junction proteins	↑ Final BW and daily weight gain; ↓ FCR; ↑ VH and VH:CD in jejunum; ↑ Expression of *Claudin-1*, *Claudin-4*, *Occludin*, *ZO-1* in jejunum
He et al. [5], United States	36; 15 gilts and 21 barrows	21 days; initial BW: 7.61 ± 0.40 kg	500 mg/kg *Bacillus subtilis* (1 × 10^9^ cfu/kg of *DSM 32540* and with ETEC challenge)	28 days	Growth performance; Diarrhea score; Intestinal morphology; Complete blood counts; Serum inflammatory markers; Gene expression; Bacterial translocation; Gut microbiota	↑ BW and ADG; ↓ Diarrhea incidence and severity; ↑ Goblet cell number and sulfomucin production in the duodenal villi; ↓ *IL-1β*, *PTGS2* expression in ileal mucosa; ↑ *MUC2* expression in jejunal mucosa; ↓ Abundance of *Lachnospiraceae*, *Peptostreptococcaceae*, *Pasteurellaceae* in the ileum
He et al. [15], United States	48	21 days; initial BW: 6.17 ± 0.36 kg	2.56 × 10^9^ cfu/kg *Bacillus subtilis* with ETEC challenge	28 days	Growth performance; Diarrhea score; Bacterial translocation; Intestinal morphology; Intestinal barrier and innate immunity; Systematic immunity; Blood cells profile	↑ Final BW at day 21 PI; ↑ ADG and feed efficiency; ↓ Diarrhea frequency and fecal β-hemolytic coliforms; ↑ Jejunal *CLDN1* expression; ↓ Ileal *IL-6* and *PTGS2* expression; ↓ White blood cell count, neutrophils, monocytes, and serum haptoglobin
He et al. [41], United States	48	21 days; initial BW: 6.17 ± 0.36 kg	2.59 × 10^9^ cfu/kg *Bacillus subtilis* with ETEC challenge	28 days	Metabolite profile in ileal mucosa and colon digesta	↑ Ileal metabolites related to mucosal repair; ↑ Polyamines, nucleotides, monosaccharides, fatty acids, and organic acids in ileum; ↑ 2-monoolein, lactic acid, and maltose in colon digesta
Hu and Kim [16], China	150, Yorkshire × Landrace × Duroc	28 days; initial BW: 7.53 ± 1.23 kg	300 g of *Bacillus subtilis* (1 × 10^9^ cfu/g; *C-3102*)	42 days	Growth performance; Nutrient digestibility; Diarrhea score; Intestinal microbiota; Excreta odor contents	↑ ADG and G:F; ↑ ATTG of DM, CP, and energy; ↓ Diarrhea scores during 7 days of post-weaning; ↓ Fecal *E. coli* counts; ↑ *Lactobacillus* counts in the intestinal microbiota; ↓ Fecal total mercaptan emissions
Hu et al. [23], China	360, Duroc × Landrace × Yorkshire	26 ± 2 days; initial BW: 7.14 ± 0.63 kg	2 × 10^9^ cfu/kg of *Bacillus subtilis* (KN-42); 4 × 10^9^ cfu/kg of *Bacillus subtilis* (KN-42); 20 × 10^9^ cfu/kg of *Bacillus subtilis* (KN-42)	28 days	Growth performance; Diarrhea incidence; Bacterial diversity; Gene expression in the feces	↑ ADG and feed efficiency in medium and high groups; ↓ Diarrhea index; ↑ Fecal bacterial diversity in medium group; ↑ Fecal *Lactobacillus* in high group; ↓ *E. coli* in the feces
Jia et al. [29], China	20 female, Landrace × Yorkshire	21 days; initial BW: 6.58 ± 0.17 kg	1 × 10^8^ cfu/*Bacillus subtilis* (ASAG 216); 1 × 10^8^ cfu/*Bacillus subtilis* (ASAG 216 with DON contamination)	21 days	Growth performance; Serum biochemical parameters; Oxidative stress indicators; Pro-inflammatory cytokines in the jejunum; mRNA expression in the jejunum; Cecal microbial population; DON and DOM-1 residues in the serum, liver, and kidney	↑ ADG and feed intake under DON challenge; ↓ Serum AST; ↓ Serum DAO, endotoxin, and PYY; ↓ Serum MDA and liver H_2_O_2_; ↑ Liver GPx and jejunal SOD activity; ↑ Jejunal *OCLN* expression; ↓ Jejunal *IL-6* and *IFN-γ*; ↑ Cecal *Faecalibacterium*; ↓ Ceal *Lactobacillus* and *E. coli* ↓ Tissue DON and DOM-1 residues
Jinno et al. [43], United States	48	21 days; initial BW: 6.17 ± 0.36 kg	500 mg/kg *Bacillus subtilis* (DSM 25841; 2.56 × 10^9^ cfu/kg with ETEC challenge)	28 days	Intestinal and fecal microbiota	↓ Fecal Chao1 index; ↑ *Ruminococcaceae*, *Veillonellaceae*, and *Bifidobacteriaceae* in jejunum; ↓ *Actinomycetaceae* in jejunum; ↑ *Lactobacillaceae* in ileum and colon; ↓ *Lachnospiraceae* in ileum; ↑ Maintenance of microbial diversity in ileum; ↑ *Bifidobacteriaceae* in colon; ↑ Preservation of beneficial bacteria compared to antibiotics
Kim et al. [39], United States	48, crossbred	Unknown; initial BW: 6.73 ± 0.77 kg	1.28 × 10^9^ cfu of *Bacillus subtilis*/kg feed, with ETEC challenge; 2.56 × 10^9^ cfu of *Bacillus subtilis*/kg feed, with ETEC challenge	19 days	Growth performance; Diarrhea score; β-hemolytic coliforms; Gut permeability; Intestinal morphology; Gene expression in intestinal mucosa	↑ ADG; ↓ Intestinal transcellular and paracellular permeability with high dose; ↑ Jejunal *CFTR*, *ZO-1*, *MUC2*, and SLC5A10 expression; ↓ Ileal IL-6 expression
Lewton et al. [36], United States	80, PIC 359 × Yorkshire	21 ± 1 days; initial BW: 7.0 ± 0.6 kg	1.48 × 10^8^ cfu/g *Bacillus subtilis* (0.5 g/kg of feed)	42 days	Growth performance; Amino acid digestibility; Gross energy and nitrogen digestibility; Colonic contents pH	↑ ADG and ADFI; ↑ AID of tryptophan, cysteine, lysine, methionine, and threonine; ↓ Nitrogen digestibility
Lewton et al. [44], United States	80	21 ± 1 days; initial BW: 7.0 ± 0.60 kg	7.35 × 10^4^ cfu/g *Bacillus subtilis*	42 days	Growth performance; Health status; Intestinal morphology; Immunological markers	↑ Jejunal VH; Ascending colon CD; ↑ Plasma IgA; ↑ Jejunal *IL-10*; ↓ Rotavirus A and C in the feces
Li et al. [33], China	24, Yorkshire × Landrace × Duroc	25 ± 2 days; initial BW: 6.80 ± 0.65 kg	10^7^ cfu *Bacillus subtilis*/kg feed	14 days	Growth performance; Serum growth hormone levels and biochemical parameters; Serum cytokines and immunoglobulins	↑ ADG and ↓ F:G; ↑ Serum total protein and albumin concentrations; ↑ IgA and IgG levels; ↑ *IL-10* ↓ IL-6 and TNF-α in the serum
Liu et al. [34], China	320, Duroc × Landrace × Yorkshire	25 days; initial BW: 7.00 ± 0.50 kg	100 mg/kg *Bacillus subtilis* (*QST713*); 200 mg/kg *Bacillus subtilis* (*QST713*)	42 days	Growth performance; Health status; Fecal SCHA concentrations; Fecal microbiota composition; Fecal gene expression; Fecal *Bacillus subtilis* QST713 quantities	↑ ADFI with low dose; ↓ Diarrhea incidence in both doses; ↓ SCFA levels in both doses; Altered β-diversity
Luise et al. [40], Italy	64	24 ± 2 days; initial BW: 7.75 kg ± 0.64 kg	1.28 × 10^6^ cfu/g feed of *Bacillus subtilis* (DSM25840)	21 days	Health status; Growth performance; Plasma metabolomics; Serum immunoglobulins; Acute phase proteins in the blood; Microbiota profile; Transcriptomic profile; Intestinal morphology, histochemistry, and immunohistochemistry	↓ Fecal score; ↑ Villus mitotic index in jejunum; ↑ Enrichment of jejunal genes related to adaptive immune response; ↓ *Enterobacteriaceae* abundance in cecum
Park et al. [25], United States	21, Large White × Landrace × Duroc	21 days; initial BW: 8.19 ± 0.77 kg	0.05% *Bacillus subtilis* (DSM 32540) with 6.7 × 108 cfu/mL K88 strain of *E.coli* on day 3	21 days	Growth performance; Fecal scores; Intestinal morphology; Ileal and cecal pH; Cecal VFA concentration	↑ VH, VH:CD in jejunum; ↑ Ileal VH; ↑ G:F; ↑ Survival rate; ↓ Frequency of diarrhea; ↓ Valeric acid concentration in cecal digesta; ↓ Ileal pH; ↓ Valeric acid concentration in cecal digesta
Sudan et al. [35], Canada	96, Landrace × Yorkshire × Duroc	N/A; initial BW: 6.4 ± 0.5 kg	2 × 10^7^ cfu *Bacillus subtilis*; 2 × 10^9^ cfu *Bacillus subtilis*	28 days	Growth performance; Diarrhea rate; Gut mucosa gene expression; Fecal microbial populations	↑ ADG and feed efficiency with low dose; ↓ Diarrhea incidence; ↓ Fecal *E. coli* and total coliform counts; ↑ Fecal lactic acid bacteria and *Bacillus* spp.; ↑ Jejunal *MUC-1*, *occludin*, and *TJP-1* expression; ↓ Jejunal *IL-8* expression
Tang et al. [31], China	72, Duroc × Landrace × Yorkshire	25 days; initial BW: 7.61 ± 0.55 kg	Low- or high-protein diet with 500 g/t *Bacillus subtilis* (DSM32315; 2 × 10^9^ cfu/g)	42 days	Growth performance; Apparent total tract digestibility; Digesta pH; Intestinal morphology; Intestinal VFA’s and VBN concentration; Intestinal microflora; mRNA gene expression	↑ ADG and ADFI; ↓ F:G in low-protein diet; ↑ ATTD of CP and ether extract in low-protein diet; ↑ DM digestibility; ↓ Ileal pH; ↑ Colonic pH in high-protein diet; ↑ Jejunal and ileal VH and VH:CD in low-protein diet; ↓ CD in jejunum; ↑ Ileal acetic acid in low-protein diet; ↑ Colonic propionic and butyric acids; ↓ Colonic volatile basic nitrogen; ↑ Ileal and colonic *Bacillus* and *Bifidobacterium* populations; ↑ mRNA expression of *ZO-1*, *Occludin*-*1*, *EGF*, *GLP-2*, *IGF-1R* in ileum
Tian et al. [28], China	160, Landrace × Large White	25 days; initial BW: 7.00 ± 0.50 kg	1 × 10^6^ cfu/g feed *Bacillus subtilis A*, *B*, or *C*	42 days	Growth performance; Diarrhea rate; Plasma cytokine levels; Intestinal morphology; Microbiota diversity; Microbial communities; Gut metabolite concentrations; mRNA levels of intestinal health-related genes	↑ ADG and ADFI in B or C; ↓ Diarrhea incidence in B and or C; ↑ IL-2 and IL-10 in B in the plasma; ↑ IL-10 in C; ↑ Colonic Butyrate and total SCFA in B; ↑ *Lactobacillus* and *Faecalibacterium* in B; ↑ mRNA expression in the colon of *ZO-1* and *occludin* in B; ↑ *ZO-1* in C in colon
Wang et al. [37], United States	264, PIC1050 × DNA600	21 ± 2 days; initial BW: N/A	0.05% *Bacillus subtilis* (*Bacillus subtilis* 747 + *Bacillus subtilis* 1999); 0.05% *Bacillus subtilis* (*Bacillus subtilis* 747 + *Bacillus subtilis* 1999) with 0.2% lactylate	42 days	Growth performance; Complete blood count; Microbial counts and composition	↑ G:F and Total white blood cell count; ↑ *Ruminococcaceae* and *S24-7* families associated with feed efficiency; ↑ Butyrate-producing taxa in the gut microbiota
Wang et al. [32], South Korea	120, Landrace × Yorkshire × Duroc	28 days; initial BW: 7.73 ± 0.75 kg	0.1% *Bacillus subtilis* (*GCB-13-001* 1 × 10^8^ cfu/kg); 0.1% *Bacillus subtilis (GCB-13-001* 1 × 10^9^ cfu/kg)	42 days	Growth performance; Nutrient digestibility; Blood profiles; Fecal microbiota; Fecal score	↑ BW and ADG; ↓ F:G; ↑ ATTD of DM and CP; ↑ Fecal *Lactobacillus* counts; ↓ Fecal *E. coli* counts and fecal score
Wan et al. [30], China	24, Duroc × Landrace × Yorkshire	24 days; initial BW: 7.67 ± 0.32 kg	10^9^ cfu/g of *Bacillus subtilis*	24 days	Growth performance; Diarrhea rate; Apparent digestibility of nutrients; Plasma xylose concentration; Intestinal morphology; Disaccharidase activities; Intestinal gene expressions; Intestinal microflora	↑ Feed efficiency; ↓ Diarrhea; ↑ VH:CD in small intestine; ↑ *Lactobacillus* in ileum; ↑ *Bacillus* in cecum

↑: Increased or elevated; ↓: Decreased or reduced; N/A: Not applicable or did not change.

### 3.3. Summary of Bacillus subtilis Intervention and Evaluated Characteristics

Across the included studies, *Bacillus subtilis* was supplemented in weaned pig diets using diverse strains, doses, and experimental designs, targeting a broad range of physiological and performance parameters as shown in Table 3. Most interventions assessed growth performance, feed efficiency, intestinal morphology, microbiota composition, and immune or metabolic responses, while several explored disease or toxin challenges to evaluate the probiotic protective effects.

Among the identified strains, *Bacillus subtilis* DSM 32315 was the most frequently tested, used in studies evaluating intestinal morphology, metabolite levels, microbiota diversity, and growth performance under varying dietary protein levels [26,31]. Other well-characterized strains included BF7658 (CG MCC 1.240) [24], WS-1 [20], MB40 [45], JATP3 [21,22], PTA-6737 [42], DSM 32540 [15,25], C-3102 [16], KN-42 [23], ASAG 216 [29], DSM 25841 [43], QST713 [34], and DSM 25840 [19]. Collectively, these studies examined not only growth and digestibility outcomes but also immune, metabolic, and microbial changes, such as plasma cytokines, short-chain fatty acid (SCFA) profiles, and intestinal barrier gene expression.

A number of studies used mixed or unspecified *Bacillus subtilis* strains. For example, *Bacillus subtilis* 747 + 1999 improved growth performance and microbial composition, especially when combined with lactylate [37], while other investigations evaluated different dosages or strain blends under standard or pathogen-challenged conditions [30,33,35,36,38,44]. Studies employing enterotoxigenic *Escherichia coli* (ETEC) or *Listeria monocytogenes* challenges [15,39,45] and toxin exposure models such as deoxynivalenol (DON) [29] provided further insight into the protective roles of *Bacillus subtilis* against intestinal stressors.

Overall, the diversity of *Bacillus subtilis* strains, experimental conditions, and measured endpoints across these studies highlights the probiotic’s multifaceted roles in modulating growth performance, gut health, immune function, and metabolic homeostasis in post-weaning pigs.

### 3.4. Summary of Quality Assessment and Risk of Bias

**Figure 2:**
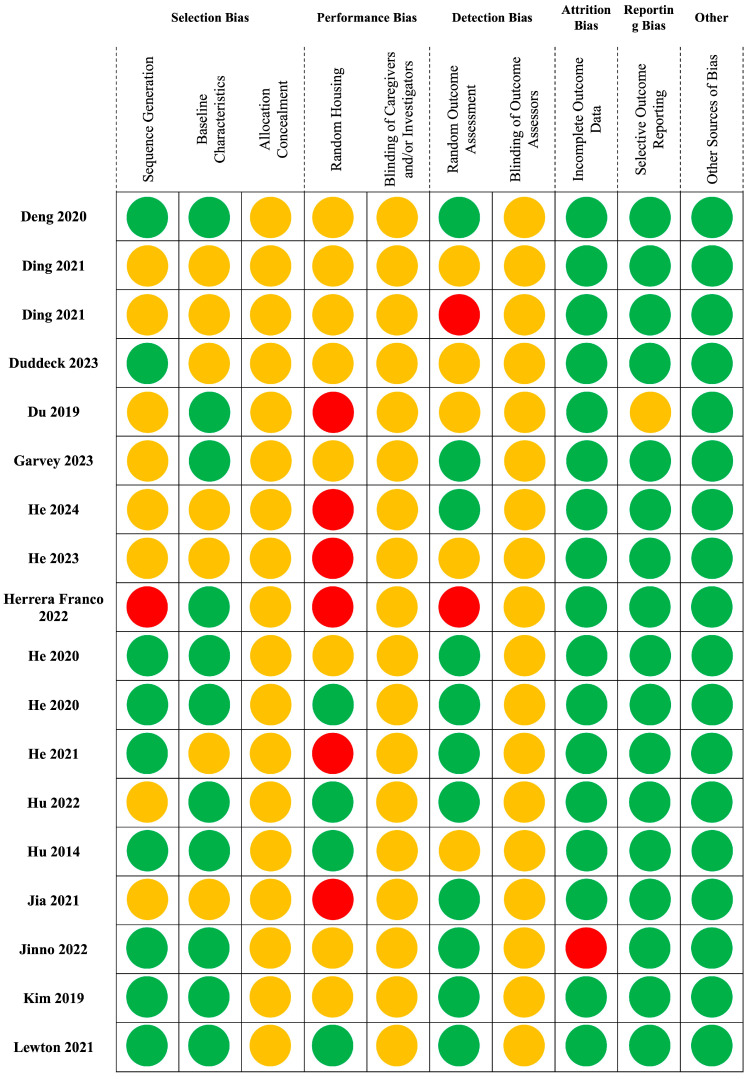

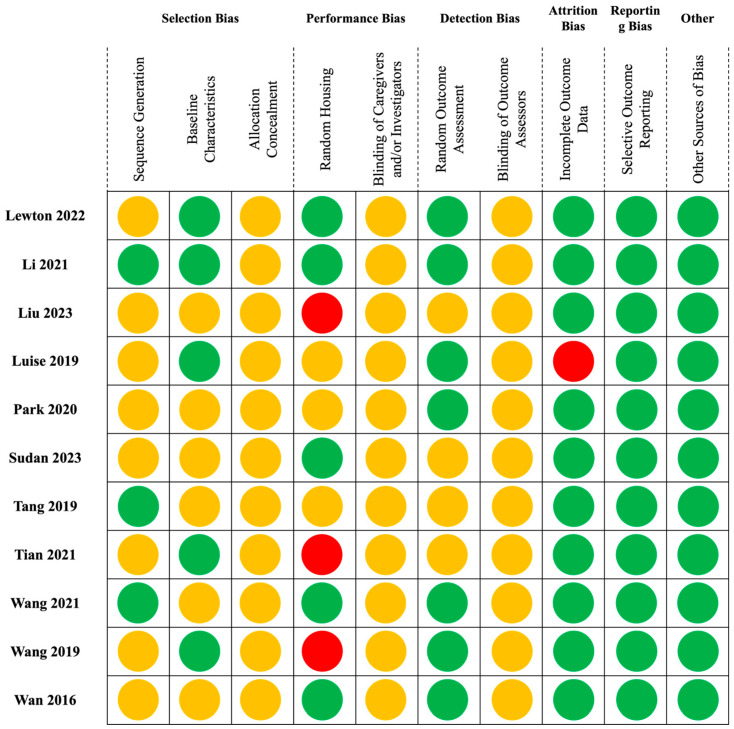
A traffic light plot of risk of bias assessments for the studies included with Green = Low risk of bias; Yellow = Unclear; Red = High risk of bias [5,15,16,20,21,22,23,24,25,26,27,28,29,30,31,32,33,34,35,36,37,38,39,40,41,42,43,44,45].

In Figure 2, the results of risk of bias assessments of the 29 studies were reported. For sequence generation, most of the studies had an unclear risk of bias, indicating possible bias in randomization [16,20,21,22,25,26,27,28,29,30,32,34,35,40,44,45]. Regarding baseline characteristics, the majority of studies had a low risk of bias, showcasing comparable baseline characteristics between groups [5,15,16,20,23,24,28,32,33,36,39,40,42,43,44,45]. Conversely, for allocation concealment, all of the studies had an unclear risk, indicating potential biases in the allocation process [5,15,16,20,21,22,23,24,25,26,27,28,29,30,31,32,33,34,35,36,37,38,39,40,41,42,43,44,45]. For random housing, there was a mixture of low, unclear, and high risks, showcasing variability between studies in whether housing was randomized. For the blinding of caregivers and/or investigators, most of the studies had an unclear risk due to a lack of sufficient information on the blinding procedures. For random outcome assessment, many of the studies reported low risk of bias [5,15,16,22,24,25,29,30,32,33,36,37,39,40,41,43,45]. Regarding blinding of outcome assessors, all the studies had an unclear risk. All studies had a low risk of incomplete outcome data bias which ensures proper handling of data acquisition and processing except for Jinno [43]. Selective outcome reporting was generally low across studies, suggesting comprehensive reporting of outcomes, except for Du [20], which was unclear. All studies had a low risk for other sources of bias, suggesting minimal other sources of bias.

The predominance of “unclear risk” ratings across several domains was primarily due to insufficient methodological reporting rather than evidence of methodological deficiencies. In particular, many studies did not clearly describe sequence generation procedures, allocation concealment, or blinding methods. These omissions limit the ability to fully assess internal validity and may reduce confidence in some reported outcomes. Future studies should follow established reporting guidelines for animal experiments to improve transparency, reproducibility, and interpretation of probiotic efficacy in swine research.

### 3.5. Growth Performance

#### 3.5.1. Body Weight (BW) and Average Daily Gain (ADG)

Across multiple studies, *Bacillus subtilis* supplementation consistently improved growth performance in weaned pigs, reflected by increases in ADG and BW. Hu et al. [23] reported that a medium dose of *Bacillus subtilis* KN-42 (4 × 10^9^ cfu/kg) increased ADG during phase 2 (days 15–28 post-weaning) compared with the negative control. Li et al. [33] observed a significant ADG increase from day 0 to 14 with 1 × 10^7^ cfu/kg feed. Similarly, Lewton et al. [36] found a tendency for higher ADG during week 2 in pigs fed 0.5 g/kg *Bacillus subtilis* (1.48 × 10^8^ cfu/g). Tang et al. [31] demonstrated that 500 g/t *Bacillus subtilis* DSM 32315 (2 × 10^9^ cfu/g) in a low-protein diet improved ADG relative to other treatments, with overall increases across the experimental period. Several other studies also showed enhanced ADG and BW responses: Wang et al. [32] reported improvements at both 1 × 10^8^ and 1 × 10^9^ cfu/kg; Hu and Kim [16] found consistent ADG increases with *Bacillus subtilis* C-3102 (1 × 10^9^ cfu/g) across all feeding phases; and Sudan et al. [35] observed higher BW and ADG in pigs receiving low-dose 2 × 10^7^ cfu during days 14–28. Tian et al. [28] reported that strains A and C (1 × 10^6^ cfu/g) increased BW on days 7 and 21, with strain C also improving final BW and ADG at day 42 compared to control.

Deng et al. [24] found that 0.1% *Bacillus subtilis* BF7658 (1 × 10^10^ cfu/g) improved final BW and ADG on day 28, while He et al. [21] reported higher BW and an upward ADG trend with 10 mL of *Bacillus subtilis* JATP3 (1 × 10^9^ cfu/mL). Herrera Franco et al. [42] observed that 50 ppm *Bacillus subtilis* PTA-6737 (10^8^ cfu) produced the highest BW and daily weight gain compared with antimicrobial-supplemented diets. Kim et al. [39] similarly demonstrated that *Bacillus subtilis* increased BW (day 5 post-inoculation) and ADG (day 0–5 and 0–11 post-inoculation) in ETEC-challenged piglets. Hu and Kim [16] further reported that 300 g of *Bacillus subtilis* C-3102 (1 × 10^9^ cfu/g) enhanced BW on days 21 and 42 and increased ADG across all sub-periods and overall. Consistently, Wang et al. [32] showed that high-dose *Bacillus subtilis* (1 × 10^9^ cfu/kg) increased BW and ADG during days 1–7 and 8–21, with sustained BW gains and higher overall ADG throughout the 42-day experiment. Overall, these findings demonstrate that supplementation of *Bacillus subtilis* at varying doses and strains consistently promotes improvements in ADG and BW in weaned pigs under both normal and challenge conditions.

#### 3.5.2. Feed Intake and Feed Efficiency

Supplementation with *Bacillus subtilis* generally improved feed efficiency and feed intake in weaned pigs, although the magnitude of response varied among studies depending on strain and inclusion level. Hu et al. [23] reported that high-dose *Bacillus subtilis* KN-42 (20 × 10^9^ cfu/kg) enhanced gain-to-feed ratio (G:F) during phase 1 compared with both negative and antibiotic controls, while medium (4 × 10^9^ cfu/kg) and low (2 × 10^9^ cfu/kg) doses also improved G:F versus the negative control. The medium and high doses further increased overall G:F across the trial. Consistent findings were observed by Wang et al. [37], where pigs fed 0.05% *Bacillus subtilis* (strains 747 + 1999) exhibited higher G:F, particularly when combined with 0.2% lactylate, and by Hu and Kim [16], who reported an overall improvement in G:F with *Bacillus subtilis* C-3102 (1 × 10^9^ cfu/g). In contrast, Lewton et al. [36] observed a reduction in G:F during week 6 in pigs supplemented with 0.5 g/kg *Bacillus subtilis* (1.48 × 10^8^ cfu/g).

Similarly, *Bacillus subtilis* lowered the feed-to-gain ratio (F:G) in several experiments. Wan et al. [30] found that 1 × 10^9^ cfu/g diet decreased F:G compared with control, while Tang et al. [31] showed that 500 g/t DSM 32315 (2 × 10^9^ cfu/g) reduced F:G, especially in low-protein diets with significant decreases during phase 2 and overall. Wang et al. [32] also reported that a high dose (1 × 10^9^ cfu/kg) significantly lowered F:G compared with the control. In terms of feed conversion ratio (FCR), Li et al. [33] observed that 1 × 10^7^ cfu/kg feed reduced FCR from day 0 to 14 relative to the negative control, while Herrera Franco et al. [42] found that 50 ppm *Bacillus subtilis* PTA-6737 (10^8^ cfu) decreased FCR compared with other treatments containing antimicrobials. Sudan et al. [35] reported that both low (2 × 10^7^ cfu) and high (2 × 10^9^ cfu) doses reduced FCR during period 2, and Tian et al. [28] observed lower FCR from days 1–7 with strains B and C (1 × 10^6^ cfu/g), with strain C also showing reduced FCR from days 8–21 versus control.

Regarding feed intake, several studies documented moderate increases following *Bacillus subtilis* supplementation. Hu and Kim [16] reported that *Bacillus subtilis* C-3102 (1 × 10^9^ cfu/g) increased average daily feed intake (ADFI) from days 8–21 and overall. Lewton et al. [36] found higher ADFI during weeks 2 and 3 in pigs fed 0.5 g/kg *Bacillus subtilis* (1.48 × 10^8^ cfu/g), while Liu et al. [13] observed an ADFI increase with 100 mg/kg *Bacillus subtilis* QST713 compared with controls. Similarly, Tang et al. [31] noted that 500 g/t DSM 32315 (2 × 10^9^ cfu/g) increased ADFI during phase 1.

The improvements in growth performance observed across many studies are likely multifactorial. Bacillus subtilis has been shown to enhance nutrient digestibility, improve intestinal morphology, and reduce post-weaning diarrhea, all of which may increase nutrient utilization and support greater weight gain. Improved villus architecture and barrier function may increase absorptive capacity, while modulation of gut microbiota may reduce competition from pathogenic microorganisms and promote fermentation profiles favorable for growth. However, responses were not uniform across studies. Variability may be explained by differences in Bacillus subtilis strain, supplementation level, feeding duration, dietary composition, health status, and challenge model. Such factors likely contribute to the inconsistent magnitude of growth responses reported in the literature.

#### 3.5.3. Digestibility

Wan et al. [30] reported increased apparent digestibility of crude ash and phosphorus with 1 × 10^9^ cfu/g *Bacillus subtilis*. Tang et al. [31] observed increased apparent digestibility of crude protein and ether extract in low-protein diets containing 500 g/t *Bacillus subtilis* DSM 32315 (2 × 10^9^ cfu/g), along with higher dry matter digestibility in both low- and high-protein diets versus other groups. Hu and Kim [16] found increased apparent total tract digestibility (ATTD) of dry matter, crude protein, and energy with *Bacillus subtilis* C-3102 (1 × 10^9^ cfu/g). Wang et al. [32] similarly reported increased ATTD of dry matter and crude protein with 0.1% *Bacillus subtilis* (1 × 10^9^ cfu/kg). Lewton et al. [36] showed higher apparent ileal digestibility of tryptophan, cysteine, lysine, methionine, and threonine with 0.5 g/kg *Bacillus subtilis* (1.48 × 10^8^ cfu/g), although nitrogen digestibility decreased. He et al. [21] reported increased apparent nitrogen availability and retention with *Bacillus subtilis* JATP3 (1 × 10^9^ cfu/mL).

#### 3.5.4. Disease Challenge Models

He et al. [15] observed that 500 mg/kg *Bacillus subtilis* DSM 32540 (1 × 10^9^ cfu/kg) in ETEC F18 infected piglets led to higher BW at days 7 and 14 post-inoculation, greater ADG from day 0–7 and 7–14 post-inoculation, and higher ADFI from day 7–14 post-inoculation versus the challenged control. He et al. [5] reported that 2.56 × 10^9^ cfu/kg *Bacillus subtilis* under ETEC challenge increased final BW at day 21 post-inoculation, ADG, and G:F from day 0–21 post-inoculation compared with the challenged positive control, while antibiotic-fed pigs showed higher BW, ADG, ADFI, and G:F at certain intervals relative to the probiotic group. Park et al. [25] found that 0.05% *Bacillus subtilis* DSM 32540 increased G:F during days 3–21 after an ETEC K88 challenge and improved survival while reducing diarrhea frequency. Kim et al. [39] noted that the high-dose *Bacillus subtilis* diet (2.56 × 10^9^ cfu/kg) decreased ADFI from day 0–5 post-inoculation compared with the negative control under ETEC F18 challenge conditions. Under a mycotoxin stressor, Jia et al. [29] showed that *Bacillus subtilis* ASAG 216 (1 × 10^8^ cfu/mL in water) reversed DON-induced reductions in ADG and feed refusal, resulting in higher ADG versus control.

### 3.6. Clinical Indicators (Diarrhea, Immune Response, and Biochemical/Oxidative Parameters)

#### 3.6.1. Diarrhea

In unchallenged piglets, *Bacillus subtilis* supplementation consistently reduced diarrhea incidence, frequency, and severity during the early post-weaning period. Sudan et al. [35] noted that the addition of 2 × 10^9^ cfu *Bacillus subtilis* saw a lower diarrhea incidence. Hu and Kim [16] reported that 300 g of *Bacillus subtilis* C-3102 (1 × 10^9^ cfu/g) decreased diarrhea scores on days 1–7 compared with the control group. Luise et al. [40] found that 1.28 × 10^6^ cfu/g feed of *Bacillus subtilis* DSM 25840 reduced fecal score impairment on day 14. Hu et al. [23] demonstrated that 2 × 10^9^, 4 × 10^9^, and 20 × 10^9^ cfu/kg of *Bacillus subtilis* KN-42 significantly lowered diarrhea incidence during both phases 1 and 2. Similarly, Tian et al. [28] observed reduced diarrhea incidence in piglets receiving 1 × 10^6^ cfu/g feed of *Bacillus subtilis* strains A, B, or C. Wan et al. [30] reported that 1 × 10^9^ cfu/g diet of *Bacillus subtilis* decreased diarrhea rate, while Liu et al. [34] found that both 100 and 200 mg/kg of *Bacillus subtilis* QST713 lowered diarrhea rates on days 1 to 21 and 1 to 42 compared with controls. Du et al. [20] also noted that pigs inoculated with *Bacillus subtilis* WS-1 (5 × 10^9^ cfu/mL) had reduced diarrhea rates on day 1 and throughout the experimental period.

In pathogen-challenged piglets, *Bacillus subtilis* also exhibited protective effects against enteric infection-induced diarrhea. He et al. [5] observed that 2.56 × 10^9^ cfu/kg *Bacillus subtilis* significantly reduced diarrhea scores from days 4 to 9 post-inoculation in piglets challenged with ETEC F18. Similarly, He et al. [15] reported that 500 mg/kg *Bacillus subtilis* DSM 32540 (1 × 10^9^ cfu/kg) mitigated diarrhea severity on days 2 to 3 post-inoculation and reduced overall diarrhea frequency. Park et al. [25] found that 0.05% *Bacillus subtilis* DSM 32540 (6.7 × 10^8^ cfu/mL) tended to lower diarrhea frequency under ETEC K88 challenge.

#### 3.6.2. Immune Response

With pigs under normal rearing conditions, *Bacillus subtilis* supplementation generally enhances immune regulation by promoting anti-inflammatory cytokines and immunoglobulin production while reducing pro-inflammatory mediators. Tian et al. [28] reported that 500 g/t *Bacillus subtilis* DSM 32315 (2 × 10^9^ cfu/g) increased plasma IL-2 and IL-10 concentrations. Similarly, Lewton et al. [44] observed a tendency for increased IL-10 levels in the jejunum and a trend toward higher plasma IgA on day 2. Li et al. [33] found that 1 × 10^7^ cfu/kg feed decreased serum IFN-γ and TNF-α while increasing IL-10 compared with the negative control. The same study also reported reduced IL-6 levels and elevated serum total protein, albumin, IgA, and IgG concentrations.

For pigs in pathogen-challenged conditions, *Bacillus subtilis* modulated inflammatory and hematological parameters, often attenuating the immune stress caused by infection. In ETEC-challenged pigs, He et al. [5] observed that 2.56 × 10^9^ cfu/kg *Bacillus subtilis* increased ileal TNF-α expression relative to an antibiotic control, indicating localized immune activation. However, in a related study, the same strain reduced total white blood cell, neutrophil, and monocyte counts and lowered serum haptoglobin concentrations compared with the positive control. Additionally, red blood cells were decreased on days 2 and 7 post-inoculation, while mean corpuscular volume and mean corpuscular hemoglobin were elevated at later time points, reflecting hematological adaptation to stress. He et al. [15] reported that 500 mg/kg *Bacillus subtilis* DSM 32540 (1 × 10^9^ cfu/kg) reduced lymphocyte counts on days 7 and 14 post-inoculation. Similarly, Garvey et al. [45] observed that 7.5 × 10^9^ cfu/day *Bacillus subtilis* reduced circulating monocyte concentrations in pigs challenged with Listeria monocytogenes, whereas control pigs showed elevated monocytes. Wang et al. [37] reported that 0.05% *Bacillus subtilis* (strains 747 + 1999) increased total white blood cell counts in non-challenged pigs, suggesting strain- and context-dependent effects on hematology.

#### 3.6.3. Gene Expression

In unchallenged piglets, *Bacillus subtilis* supplementation consistently modulated intestinal gene expression related to barrier integrity, nutrient absorption, and immune regulation. Sudan et al. [35] reported that supplementation with 2 × 10^9^ cfu *Bacillus subtilis* reduced intestinal *IL-8* mRNA levels, while increasing the expression of *MUC1*, *occludin*, and *TJP-1* compared with the negative control. Tang et al. [31] observed that 500 g/t *Bacillus subtilis* (DSM32315; 2 × 10^9^ cfu/g) increased the mRNA expression of *ZO-1*, *occludin*, *EGF*, *GLP-2*, and *IGF-1R* in the ileum. Similarly, He et al. [22] found that *Bacillus subtilis* JATP3 (1 × 10^9^ cfu/mL) increased the relative expression of *claudin*, *ZO-1*, and *IL-10* while decreasing *IL-1β* in both the jejunum and ileum. Herrera Franco et al. [42] reported that 50 ppm of *Bacillus subtilis* PTA-6737 (10^8^ cfu) enhanced mRNA expression of tight junction proteins, including *claudin-1*, *claudin-4*, *occludin*, and *ZO-1* in the jejunum. Likewise, Liu et al. [34] observed significant upregulation of *occludin* expression with 100 mg/kg of *Bacillus subtilis*. Luise et al. [40] found that 1.28 × 10^6^ cfu/g *Bacillus subtilis* DSM25840. Collectively, these results indicate that *Bacillus subtilis* enhances intestinal barrier function and epithelial immune defense under normal conditions through the upregulation of tight junction and immune-related genes.

In pathogen or toxin-challenged piglets, *Bacillus subtilis* also modulated gene expression associated with inflammation and mucosal defense. In ETEC-infected pigs, He et al. [15] reported that 500 mg/kg *Bacillus subtilis* DSM 32540 (1 × 10^9^ cfu/kg) upregulated *MUC2* expression in the jejunal mucosa while downregulating *PTGS2* and *IL-1β* in the ileal mucosa. He et al. [5] found that 2.56 × 10^9^ cfu/kg *Bacillus subtilis* increased *CLDN1* expression in the jejunal mucosa and reduced *IL-6* and *PTGS2* expression in the ileum compared with the positive control. Similarly, Kim et al. [39] observed that 2.56 × 10^9^ cfu/kg *Bacillus subtilis* increased *CFTR* and *ZO-1* mRNA expression on day 5 post-inoculation and *SLC5A10* on day 11 post-inoculation, while *MUC2* expression was modulated over time, which decreased on day 5 post-inoculation but increased on day 11 post-inoculation compared with the positive control. Jia et al. [29] reported that in DON-contaminated piglets, *Bacillus subtilis* ASAG 216 (1 × 10^8^ cfu/mL) alleviated oxidative stress by decreasing *SOD3* and increasing *GPX2* and *NOS2* expression, while restoring *OCLN* expression in the jejunum affected by DON exposure.

Overall, *Bacillus subtilis* supplementation upregulated genes associated with epithelial integrity (*ZO-1*, *Claudins*, *Occludin*, *MUC2*), growth and repair (*EGF*, *IGF-1R*, *GLP-2*), and anti-inflammatory signaling (*IL-10*, *GPX2*, *NOS2*) under both normal and challenged conditions. The consistent upregulation of genes associated with tight junction integrity and mucosal defense suggests that maintenance of intestinal barrier function may be one of the primary mechanisms through which *Bacillus subtilis* supports pig health. However, the magnitude of transcriptional responses differed among studies, likely due to variation in strain characteristics, intestinal sampling location, health challenge status, and supplementation duration.

#### 3.6.4. Biochemical/Oxidative Parameters

In unchallenged piglets, *Bacillus subtilis* supplementation influenced multiple biochemical and metabolic indicators associated with energy metabolism, fermentation, and amino acid utilization. Tang et al. [31] reported that a diet containing 500 g/t *Bacillus subtilis* DSM32315 (2 × 10^9^ cfu/g) with low protein increased ileal acetic acid concentrations, while colonic propionic and butyric acid levels also increased in both low- and high-protein diets. Deng et al. [24] found that supplementation with 0.1% *Bacillus subtilis* (F7658) (1 × 10^10^ cfu/g) elevated serum glucose and triglyceride concentrations. Similarly, Duddeck et al. [38] observed that diets containing 3.75 × 10^8^ cfu/g or 3.75 × 10^9^ cfu/g *Bacillus subtilis* increased blood glucose levels at day 14 post-weaning compared with the control. He et al. [21] reported that supplementation of 10 mL of JATP3 (1 × 10^9^ cfu/mL) elevated α-ketoglutarate (AKG) content in liver and muscle, as well as plasma AKG and L-lactic acid levels, while reducing plasma indole concentrations. The same study observed increased phosphorylation of 4EBP1 in skeletal muscle, indicating enhanced protein synthesis signaling. In contrast, Liu et al. [34] reported that 100 mg/kg *Bacillus subtilis* QST713 decreased butyrate levels and reduced acetate, butyrate, valerate, and total SCFA concentrations. 200 mg/kg *Bacillus subtilis* QST713 also decreased acetate and total SCFA levels, suggesting possible dose-dependent modulations of microbial fermentation.

In pathogen- or toxin-challenged piglets, *Bacillus subtilis* exerted protective effects by enhancing antioxidant defense systems and mitigating oxidative damage. Jia et al. [29] found that in DON-contaminated piglets, inclusion of *Bacillus subtilis* ASAG 216 (1 × 10^8^ cfu/mL) increased the activities of GPx and SOD, while decreasing MDA and H2O2 concentrations that were elevated by DON exposure. These results suggest that *Bacillus subtilis* can attenuate oxidative stress and restore redox balance in toxin-challenged pigs.

The reductions in diarrhea incidence and improvements in immune-related outcomes suggest that Bacillus subtilis may contribute to improved post-weaning resilience through multiple pathways. Proposed mechanisms include competitive exclusion of enteric pathogens, enhancement of mucosal barrier integrity, and modulation of inflammatory signaling. Nevertheless, responses varied among studies, particularly for biochemical and oxidative stress markers. This inconsistency may reflect differences in strain-specific probiotic activity, challenge severity, sampling time points, and analytical methods used to quantify immune and metabolic responses.

### 3.7. Intestinal Development

In healthy piglets, *Bacillus subtilis* supplementation consistently enhances intestinal morphology, particularly by improving villus architecture and epithelial maturation across intestinal segments.

In the ileum, Deng et al. [24] reported that supplementation with 0.1% *Bacillus subtilis* BF7658 (CGMCC 1.240; 1 × 10^10^ cfu/g) significantly increased villus height (VH) and the villus height-to-crypt depth ratio (VH:CD) compared with the control. Similarly, Ding et al. [26] observed increased ileal VH and VH:CD in pigs fed 500 g/t *Bacillus subtilis* DSM 32315 (2 × 10^9^ cfu/g), while Tian et al. [28] reported that supplementation with *Bacillus subtilis* strains A, B, or C (1 × 10^6^ cfu/g) improved ileal VH and VH:CD.

Improvements were also evident in the jejunum. Herrera Franco et al. [42] reported increased jejunal VH and VH:CD following supplementation with 50 ppm *Bacillus subtilis* PTA-6737 (10^8^ cfu). Lewton et al. [44] similarly observed increased jejunal villus height with 7.35 × 10^4^ cfu/g *Bacillus subtilis*. Tang et al. [31] found that pigs fed a low-protein diet containing 500 g/t *Bacillus subtilis* DSM 32315 exhibited increased jejunal VH:CD and reduced crypt depth (CD), indicating improved absorptive capacity.

Across multiple intestinal segments, Wan et al. [30] reported that supplementation with 1 × 10^9^ cfu/g *Bacillus subtilis* increased VH:CD in the duodenum, jejunum, and ileum, while decreasing jejunal CD compared with the control. Tang et al. [31] further demonstrated that pigs fed a low-protein diet with *Bacillus subtilis* had the greatest villus height overall. Additional indicators of intestinal development were reported by He et al. [22], who found that supplementation with *Bacillus subtilis* JATP3 (1 × 10^9^ cfu/mL) increased the villus gland ratio and decreased jejunal and ileal pH. Luise et al. [40] also observed an increased villus mitotic index in pigs fed 1.28 × 10^6^ cfu/g *Bacillus subtilis* DSM 25840, suggesting enhanced epithelial cell proliferation.

In disease-challenged piglets, *Bacillus subtilis* supplementation modulated intestinal morphology and barrier integrity, although responses varied depending on challenge conditions and comparator treatments. In pigs challenged with ETEC K88, Park et al. [25] reported that supplementation with 0.05% *Bacillus subtilis* DSM 32540 increased jejunal VH and VH:CD and ileal VH, with effects exceeding those observed in antibiotic-treated pigs. Kim et al. [39] found that supplementation with 2.56 × 10^9^ cfu/kg *Bacillus subtilis* reduced jejunal permeability on days 5 and 11 post-inoculation compared with the positive control, and that both low and high doses increased sialomucin area in duodenal crypts on day 5 post-inoculation.

He et al. [15] reported that supplementation with 500 mg/kg *Bacillus subtilis* DSM 32540 (1 × 10^9^ cfu/kg) in ETEC-challenged piglets increased ileal VH, CD, goblet cell number, and sulfomucin production in the duodenum, indicating enhanced mucosal protection. In contrast, He et al. [5] observed that piglets receiving 2.56 × 10^9^ cfu/kg *Bacillus subtilis* under ETEC challenge had reduced duodenal VH, sialomucin percentage, and crypt area compared with the antibiotic group, suggesting context-dependent responses influenced by comparator treatments.

In a toxin-challenge model, Jia et al. [29] reported that supplementation with *Bacillus subtilis* ASAG 216 (1 × 10^8^ cfu/mL) in DON-contaminated piglets reduced serum diamine oxidase, endotoxin, and peptide YY concentrations, counteracting DON-induced intestinal damage. Additionally, DON and DOM-1 residues were significantly reduced across tissues in pigs receiving *Bacillus subtilis* compared with positive controls, indicating improved intestinal barrier integrity and detoxification capacity.

Overall, these findings demonstrate that *Bacillus subtilis* supplementation enhances intestinal development and epithelial integrity in unchallenged piglets and provides protective or restorative effects on intestinal morphology and barrier function under pathogenic or toxin-induced stress. Improvements in villus height and villus height-to-crypt depth ratio may partially explain the enhanced growth performance reported in many studies. Increased absorptive surface area can improve nutrient utilization and digestive efficiency, thereby supporting greater body weight gain. However, not all studies observed improvements in intestinal morphology, suggesting that responses may depend on factors such as strain selection, dosage, dietary composition, and the physiological status of the pigs.

### 3.8. Gut Microbiota

Across studies, *Bacillus subtilis* supplementation altered gut microbial composition and related fermentation outputs, generally shifting communities toward greater abundance of beneficial taxa and reduced markers of enteric pathogens, although effects varied by strain, dose, and challenge conditions. In unchallenged piglets, Sudan et al. [35] reported that 2 × 10^9^ cfu *Bacillus subtilis* reduced fecal *E. coli* and total coliform counts while increasing lactic acid bacteria and *Bacillus* spp. Hu et al. [23] similarly observed dose-responsive increases in fecal *Lactobacillus* and reductions in fecal *E. coli* with *Bacillus subtilis* KN-42, and reported higher species richness in the medium- and high-dose groups. Wang et al. [32] found increased *Lactobacillus* counts and decreased *E. coli* counts at the end of the study in pigs fed 0.1% *Bacillus subtilis* GCB-13-001 (1 × 10^9^ cfu/kg), while Hu and Kim [16] noted a tendency for decreased *E. coli* and increased *Lactobacillus* with *Bacillus subtilis* C-3102 (1 × 10^9^ cfu/g), along with a tendency to reduce total mercaptan emissions. Several studies also reported changes in microbial diversity and community structure: Deng et al. [24] reported increased Chao-1 and a tendency for increased Shannon and Simpson indices compared with the antibiotic group, alongside increased colonic *Firmicutes* and decreased fecal *E. coli*. Ding et al. [27] observed increased microbial diversity and altered bacterial abundances with *Bacillus subtilis* DSM 32315, accompanied by increased concentrations of microbial metabolites including butyrate, tryptamine, and cadaverine, and reduced skatole.

In addition to compositional shifts, *Bacillus subtilis* influenced fermentation products and related biochemical indices. Duddeck et al. [38] reported that the low-dose *Bacillus subtilis* diet (3.75 × 10^8^ cfu/g; 1.875 × 10^5^ cfu/g diet) increased fecal butyrate and isovalerate, as well as acetate, propionate, and total SCFAs compared with the high-dose group, and detected differences in beta diversity between low- and high-dose groups at day 14. The same study noted a tendency for reduced glycohyocholic acid proportion on day 14 in both *Bacillus subtilis* groups and an increased hyodeoxycholic acid:hyocholic acid ratio with the higher dose at day 14, suggesting dose-dependent effects on bile acid profiles. Tang et al. [31] reported increased ileal *Bacillus* and *Bifidobacterium* populations in both low-protein and high-protein diets containing *Bacillus subtilis* DSM 32315 and increased colonic *Bacillus* and *Bifidobacterium* in the low-protein conditions. Park et al. [25] reported reduced valeric acid concentration in cecal digesta in pigs receiving *Bacillus subtilis* DSM 32540 under ETEC K88 challenge. He et al. [22] further reported metabolite shifts including increased isobutyric acid and isocitric acid and an association between citalopram levels and microbial changes in the jejunum and ileum.

Several studies provided detailed segment-specific microbiota changes. In the jejunum and ileum, He et al. [22] and He et al. [21] reported that *Bacillus subtilis* JATP3 supplementation (1 × 10^9^ cfu/mL) altered phylum-level and genus-level composition, including shifts in *Firmicutes*, *Bacteroidota*/*Bacteroidetes*, *Proteobacteria*, and multiple genera associated with carbohydrate fermentation and lactic acid production, although the direction of specific phylum-level changes differed between the two studies. Wan et al. [30] reported increased *Lactobacillus* in the ileum and increased *Bacillus* in the cecum, while Luise et al. [40] observed decreased *Enterobacteriaceae* abundance in the cecum following *Bacillus subtilis* DSM 25840 supplementation. Wang et al. [37] reported enrichment of taxa associated with feed efficiency (including unclassified *Ruminococcaceae* and *S24-7*) and increased butyrate-producing taxa in pigs fed *Bacillus subtilis* 747 + 1999.

In disease-challenged models, *Bacillus subtilis* supplementation also modulated microbial populations and related metabolites in ways consistent with reduced pathogen burden and improved resilience. He et al. [15] reported reduced relative abundance of pathogenic bacterial families in the ileum (including *Lachnospiraceae*, *Peptostreptococcaceae*, and *Pasteurellaceae*) in ETEC-challenged piglets supplemented with *Bacillus subtilis* DSM 32540. He et al. [41] reported increases in metabolites linked to mucosal energy and repair (e.g., 2-monoolein, lactic acid, and maltose) in ileal mucosa and colon digesta of ETEC-challenged pigs receiving *Bacillus subtilis* compared with antibiotic controls. Jia et al. [29] found that under DON contamination, *Bacillus subtilis* ASAG 216 altered cecal microbial composition, including increased *Faecalibacterium* and reduced *Escherichia*, along with reductions in other taxa impacted by DON exposure.

Overall, the included studies indicate that *Bacillus subtilis* can reshape gut microbial communities and metabolic outputs in weaned pigs, frequently increasing taxa associated with gut health (e.g., *Lactobacillus*, butyrate-related communities) while reducing indicators of enteric pathogens (e.g., *E. coli*/coliforms), with additional modulation of SCFA production, bile acid profiles, and community diversity. Although the specific microbial responses varied considerably among studies, a common pattern was the enrichment of bacterial taxa generally associated with intestinal health and the reduction in potentially pathogenic microorganisms. These microbial shifts may contribute to improved nutrient utilization, enhanced barrier function, and reduced inflammation. However, microbiota outcomes were among the most heterogeneous findings in this review, likely reflecting differences in sequencing methodologies, intestinal sampling sites, probiotic strains, dietary formulations, and environmental conditions.

## 4. Strengths and Limitations

A major strength of this systematic review is its comprehensive evaluation of the effects of *Bacillus subtilis* supplementation across a wide range of outcomes in weanling piglets, including growth performance, clinical indicators, gene expression, biochemical and oxidative parameters, intestinal development, and gut microbiota composition. By synthesizing evidence across 29 studies, this review provides a broad overview of how different strains, dosages, dietary conditions, and challenge models may influence the responses to *Bacillus subtilis* supplementation. Many included studies reported improvements in average daily gain, body weight, diarrhea incidence, intestinal morphology, immune regulation, and gut microbial composition, suggesting that *Bacillus subtilis* has potential as a functional feed additive during the post-weaning period.

However, several limitations should be acknowledged. First, this review was restricted to English-language, peer-reviewed, full-text publications, which may have introduced language and publication bias and may have excluded relevant studies published in other languages or in non-peer-reviewed formats. Second, dissertations, conference abstracts, and other forms of gray literature were excluded because they often lack sufficient methodological detail for consistent data extraction and risk of bias assessment; however, this may have reduced the comprehensiveness of the evidence base. Third, substantial heterogeneity was present among the included studies, including differences in *Bacillus subtilis* strain, dosage, supplementation duration, dietary composition, piglet age, comparator treatments, health challenge models, sampling time points, and outcome measurements. These differences limited direct comparisons across studies and contributed to variability in reported outcomes.

In addition, a quantitative meta-analysis was not performed because of heterogeneity in study designs and inconsistent reporting of comparable outcome data, including variance measures and standardized units. Therefore, the conclusions of this review are based on qualitative syntheses and should be interpreted as evidence of general trends rather than pooled effect estimates. Studies that evaluated *Bacillus subtilis* in combination with other direct-fed microbial species were also excluded because their outcomes could not be directly attributed to *Bacillus subtilis* alone. Finally, most included studies were short-term, commonly lasting 28 days or less, limiting conclusions regarding long-term effects on growth performance, gut health, immune response, and metabolic regulation. Future studies using standardized designs, longer feeding durations, consistent outcome measures, and mechanistic approaches are needed to improve confidence in practical recommendations for *Bacillus subtilis* supplementation in weanling piglets.

## 5. Conclusions

This systematic review indicates that supplementation with *Bacillus subtilis* can improve growth performance, gut health, and intestinal development in weanling piglets, with many studies reporting increased average daily gain and body weight during the critical post-weaning period. Several strains also demonstrated the ability to modulate gut microbiota, enhance intestinal morphology, and support immune function, contributing to improved nutrient utilization. However, responses varied across studies, highlighting the importance of strain selection, dosage, dietary context, and experimental design. In addition, the limited duration of most studies restricts conclusions regarding long-term effects and underlying mechanisms. Overall, *Bacillus subtilis* shows promise as a nutritional strategy for weanling piglets, but further long-term and mechanistic research is needed to optimize its application in swine production systems.

## Figures and Tables

**Table 1:** Summary of inclusion and exclusion criteria.

	Inclusion Criteria	Exclusion Criteria
Population (P)	Pigs (*Sus domesticus* and/or *sus scrofa*); Breeds: Commonly used in the industry	Non-pig species (e.g., guinea pig)
Intervention (I)	Dietary supplementation with various *Bacillus subtilis* strains and dosages	*Bacillus subtilis* combined with other treatments
Comparators (C)	A control group fed a commercial or basal diet not containing *Bacillus subtilis*; Extra comparators: Different concentrations or strains of *Bacillus subtilis* and combinations in both feed and water	Studies not containing a control group
Outcomes (O)	Growth performance; Incidence and severity of diarrhea; Overall health status; Feed preference and palatability; Health of gut and microbiota composition; Development of intestine; or Blood biochemical parameters	Studies with measurements only in vitro or ex vivo
Study Designs (S)	Controlled experimental trials including random allocation into control and treatment groups	Observational studies including cross-sectional, case–control, cohort studies, lacking a clear intervention, detailed methodology, or outcomes measurement procedures

**Table 2:** Search strategies for PubMed, Scopus, and Agricola.

**PubMed**	
Query	(“*Bacillus subtilis*”[All Fields] OR “B. subtilis”[All Fields] AND (“Piglet”[All Fields] OR “Piglets”[All Fields] OR “Pig”[All Fields] OR “Pigs”[All Fields] OR “Boar”[All Fields] OR “Swine”[All Fields] OR “Gilt”[All Fields] OR “Porcine”[All Fields] OR “Sus scrofa”[All Fields]) AND (2000/1/1:2025/12/12[pdat])
Language	Limited by English
Range	Year 2000 to 2025
**Scopus**	
Query	bacillus AND subtilis OR b. AND subtilis AND piglet OR piglets OR pig OR pigs OR swine OR porcine OR gilt OR boar OR sus AND scrofa AND PUBYEAR > 1999 AND PUBYEAR < 2026 AND (LIMIT-TO (LANGUAGE, “English”)) AND (LIMIT-TO (EXACTKEYWORD, “Pig”) OR (LIMIT-TO (EXACTKEYWORD, “Swine”))
Language	Limited by English
Range	Year 2000 to 2025
**Agricola**	
Query	bacillus AND subtilis OR b. AND subtilis AND piglet OR piglets OR pig OR pigs OR swine OR porcine OR gilt OR boar OR sus AND scrofa
Language	Limited by English
Range	Year 2000 to 2025

## Data Availability

No new datasets were generated or analyzed during this study. All data supporting the findings of this systematic review are derived from previously published studies cited within the article. The review protocol is publicly available through the Open Science Framework (OSF) Registries (ID: vx7d3).

## Abbreviations

The following abbreviations are used in this manuscript:

ADFI	average daily feed intake
ADG	average daily gain
AKG	α-ketoglutarate
ATTD	apparent total tract digestibility
BW	body weight
CD	crypt depth
cfu	colony-forming units
CFTR	cystic fibrosis transmembrane conductance regulator
CLDN1	claudin-1
DON	deoxynivalenol
DOM-1	deepoxy-deoxynivalenol
*E. coli*	*Escherichia coli*
EGF	epidermal growth factor
ETEC	enterotoxigenic *Escherichia coli*
F:G	feed-to-gain ratio
FCR	feed conversion ratio
G:F	gain-to-feed ratio
GLP-2	glucagon-like peptide-2
GPx	glutathione peroxidase
GPX2	glutathione peroxidase 2
H2O2	hydrogen peroxide
IFN-γ	interferon-gamma
IgA	immunoglobulin A
IgG	immunoglobulin G
IGF-1R	insulin-like growth factor 1 receptor
IL	interleukin
MDA	malondialdehyde
mRNA	messenger ribonucleic acid
MUC1	mucin 1
MUC2	mucin 2
NOS2	nitric oxide synthase 2
OCLN	occludin
PICOS	populations, interventions, comparators, outcomes, and study designs
PI	post-inoculation
PRISMA	Preferred Reporting Items for Systematic Reviews and Meta-Analyses
PTGS2	prostaglandin-endoperoxide synthase 2
SCFA	short-chain fatty acid
SLC5A10	solute carrier family 5 member 10
SOD	superoxide dismutase
SOD3	superoxide dismutase 3
spp.	species
TJP-1	tight junction protein 1
TNF-α	tumor necrosis factor-alpha
VH	villus height
ZO-1	zonula occludens-1
